# Enzyme linked oligonucleotide assay for the sensitive detection of SARS-CoV-2 variants

**DOI:** 10.3389/fcimb.2022.1017542

**Published:** 2022-09-29

**Authors:** Michael Shola David, Damira Kanayeva

**Affiliations:** Department of Biology, School of Sciences and Humanities, Nazarbayev University, Nur-Sultan, Kazakhstan

**Keywords:** SARS-CoV-2, RBD, spike protein, aptamer, ELONA, detection, virus

## Abstract

The exponential spread of COVID-19 has prompted the need to develop a simple and sensitive diagnostic tool. Aptamer-based detection assays like ELONA are promising since they are inexpensive and sensitive. Aptamers have advantages over antibodies in wide modification, small size, *in vitro* selection, and stability under stringent conditions, which aid in scalable and reliable detection. In this work, we used aptamers against SARS-CoV-2 RBD S protein to design a simple and sensitive ELONA detection tool. Screening CoV2-RBD-1C and CoV2-RBD-4C aptamers and optimizing assay conditions led to the development of a direct ELONA that can detect SARS-CoV-2 RBD S glycoprotein in buffer solution and 0.1 % human nasal fluid with a detection limit of 2.16 ng/mL and 1.02 ng/mL, respectively. We detected inactivated Alpha, Wuhan, and Delta variants of SARS-CoV-2 with the detection limit of 3.73, 5.72, and 6.02 TCID_50_/mL, respectively. Using the two aptamers as capture and reporter elements, we designed a more sensitive sandwich assay to identify the three SARS-CoV-2 variants employed in this research. As predicted, a lower detection limit was obtained. Sandwich assay LOD was 2.31 TCID_50_/mL for Alpha, 1.15 TCID_50_/mL for Wuhan, and 2.96 TCID_50_/mL for Delta. The sensitivity of sandwich ELONA was validated using Alpha and Wuhan variants spiked in 0.1% human nasal fluid sample condition and were detected in 1.41 and 1.79 TCID_50_/mL LOD, respectively. SEM was used to visualize the presence of viral particles in the Delta variant sample. The effective detection of SARS-CoV-2 in this study confirms the potential of our aptamer-based technique as a screening tool.

## Introduction

In late January 2020, coronavirus was declared public health emergency of international concern by the (www.who.int, WHO, 2020). As of September 7 2022, 6,507,589 people have died from the severe complication associated with severe acute respiratory syndrome coronavirus 2 (SARS-CoV-2). SARS-CoV-2 may lead to some severe health problems notably in patients who are immunocompromised or who have a history of underlying medical conditions (Lobanova, 2022).

SARS-CoV-2 infection is thought to be mediated by the spike (S) glycoprotein, which is the key component that triggers fusion with the host receptor and plays an important role in the virus’s pathogenicity (Hopf et al., 2014; Pontes et al., 2021). A large number of these glycosylated proteins help the virus evade the immune response and protect the surface of the virus from neutralizing antibodies (V’kovski et al., 2021). S proteins are functionally divided into two subunits namely S1 and S2. The S1 subunit which is exposed to the surface of the virus is composed of the receptor-binding domain (RBD) that plays an important role in the recognition of the host receptor (Huang et al., 2020; V’kovski et al., 2021). The S2 subunit on the other hand is a transmembrane protein domain that consists of repeated heptad regions and peptides responsible for viral fusion and mediates the viral particle binding to a host cell membrane (V’kovski et al., 2021). This research study reports the detection of the RBD S protein, which is the major target that mediates infection in its host.

The validity of diagnostic procedures in their general adoption is mostly determined by the amount of time required to obtain results, the type of test, test’s accuracy, and the resources required for testing. The reverse transcription-polymerase chain reaction (RT-PCR) is the gold standard and is widely accepted for the detection of SARS-CoV-2 (Bustin et al., 2005; Waggoner et al., 2020). On the other hand, enzyme-linked oligonucleotide assay (ELISA) permits the sensitive and specific quantitative/qualitative identification of SARS-CoV-2 based on antigen-antibody reactions (Sakamoto et al., 2018). However, the reliance of an ELISA on antibodies exhibits some drawbacks like the batch-to-batch inconsistency, costly and time-consuming technique in the production of antibodies, and the unstable nature of antibodies during transport and storage (Toh et al., 2015). These drawbacks necessitate the need for an alternative to antibodies to enhance the ELISA method by using molecular recognition element (MRE) known as aptamers (Toh et al., 2015) resulting in an assay known as an enzyme-linked oligonucleotide assay (ELONA). ELONA is an analog of ELISA that was first developed in 1971 (Drolet et al., 1996). The ELONA method utilizes a specific aptamer commonly referred to as artificial antibodies (Wang et al., 2016). ELONA provides a simple and quick way to assess and identify a wide range of analytes with high sensitivity and specificity (Drolet et al., 1996).

**Figure 1:**
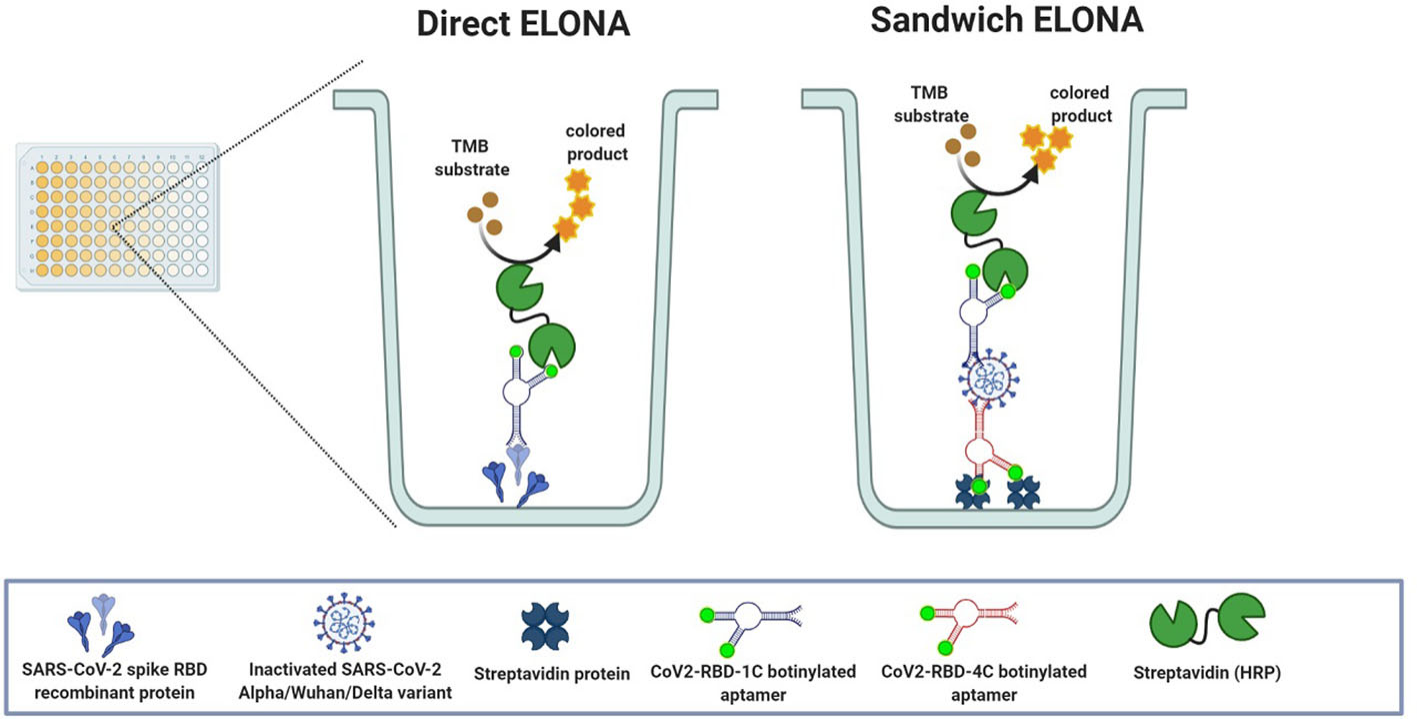
Schematic representation of both direct and sandwich ELONA.

In this study, we employed CoV2-RBD-1C and CoV2-RBD-4C aptamers that were previously selected using an ACE2 competition-based selection strategy and a machine learning screening algorithm (Song et al., 2020). These aptamer sequences were employed to develop a simple and sensitive ELONA (**Figure 1**) for the detection of SARS-CoV-2 RBD S protein in buffer solution and spiked in a 0.1% human nasal fluid. The sensitivity of the direct ELONA method was investigated by using heat-inactivated Alpha, Wuhan, and Delta variants of SARS-CoV-2 propagated in Vero E6 cells. To further improve the sensitivity of our detection method, we designed a sandwich ELONA in which we successfully improved the absorbance (OD_450_) and obtained a lower limit of detection (LOD) compared to the direct ELONA format. To validate the practicality of the sandwich ELONA method to samples in a clinical setting, both inactivated Alpha as well as Wuhan variants of SARS-CoV-2, were also spiked in 0.1% human nasal fluid. As shown in the result section, different viral titers were tested and the absorbance indicates the corresponding OD_450_ for each viral titer tested. Overall we found out that the results obtained were in good accordance with what was obtained in the literature and to the best of our knowledge, this is the first study where the heat-inactivated Alpha, Wuhan, and Delta variants of SARS-CoV-2 propagated in Vero E6 cells were detected both in buffer solution as well as in human nasal fluid using both direct and sandwich ELONA method, in which an improved sensitivity absorbance was obtained.

## Materials and method

### ELONA protocol

**Figure 1** shows the direct and sandwich ELONA approach for SARS-CoV-2 detection. In direct ELONA, the target molecule (S glycoprotein) is immobilized on a microtiter plate and forms a complex with the aptamer (CoV2-RBD-1C). With the help of an enzyme conjugate (s-HRP) reacting with the aptamer and TMB substrate, a color change was produced. Sandwich ELONA employs the same approach, but the CoV2-RBD-4C aptamer was immobilized on a streptavidin-coated 96-well plate to capture SARS-CoV-2. After adding the reporter aptamer (CoV2-RBD-1C), the remaining steps were the same as in the direct ELONA. Color intensity indicates signal readout that represents the level of aptamer reacting with the target. The method in detail is described below. ELONA schematic (**Figure 1**) was created using BioRender.com (GC2408DSS7).

### Proteins

SARS-CoV-2 (2019-nCoV) spike RBD recombinant protein (40592-VNAH), SARS-CoV-2 spike antibody chimeric MAb (40150-D005), MERS-CoV recombinant spike protein (40071-V08B1), and SARS-CoV-2 nucleocapsid-His (N) recombinant protein (40588-V08B) were purchased from Sino Biological (China). Influenza hemagglutinin (HA) peptide (≥ 97% HPLC) (12149-5MG) was purchased from Sigma-Aldrich (USA). Native human serum albumin (HSA) (ab205808) was purchased from Abcam (UK).

### Aptamers

The aptamer sequences (**Table 1**) were adopted from Song et al. (2020), which were modified with a biotin tag at the 5’ - terminus and were synthesized by Eurogentec (Belgium).

**Table 1:** SARS-CoV-2 RBD specific aptamer sequences.

Aptamer name	Sequence (5’ – 3’)
CoV2-RBD-1C	CAGCACCGACCTTGTGCTTTGGGAGTGCTGGTCCAAGG GCGTTAATGGACA
CoV2-RBD-4C	ATCCAGAGTGACGCAGCATTTCATCGGGTCCAAAAGGGGCTGCTCGGGATTGCGGATATGGACACGT

### Inactivated SARS-CoV-2 variants

Heat-inactivated SARS-CoV-2 variants (BSL-1) propagated in Vero E6 cells: i) hCoV-19/Kazakhstan/KazNAU-NSCEDI-Kaissar/2021 GISAID accession no. EPI_ISL_2349552 (Alpha variant with D614G, D1118H, H69del, N501Y, P681H, S982A, T716I, V70del, Y144del mutations in S protein; viral titer of 5.32 ± 0.13 Log_10_ TCID_50_/mL), ii) hCoV-19/Kazakhstan/KazNAU-NSCEDI-4635/2020 GISAID accession no. EPI_ISL_1208952 (Wuhan variant; with D614G and M153T mutations in the S protein; viral titer of 6.20 ± 0.00 Log_10_ TCID_50_/mL), and iii) hCoV-19/Kazakhstan/KazNARU-NSCEDI-5526/2021 GISAID accession no. EPI_ISL_4198501 (Delta variant with D614G, D950N, E156G, F157del, G142D, H1083Y, L452R, P681R, R158del, T19R, T478K mutations in S protein; viral titer of 6.45 ± 0.16 Log_10_ TCID_50_/mL) were obtained from the Masgut Aikimbayev National Scientific Center for Dangerous Infections (Almaty, Kazakhstan).

### Biotinylation of SARS-CoV-2 S antibody

SARS-CoV-2 spike antibody, chimeric MAb was biotinylated using a biotinylation kit/biotin conjugation kit (fast, type A) – lightning-link^®^ (Abcam, ab201795) according to the manufacturer’s protocol. In brief, 1 μL of a modifier reagent in the kit was added to each 10 μL of antibody to be labeled and mixed gently. Then, the antibody with the modifier reagent was added to the biotin conjugation mix found in the kit. Thereafter, the mix was gently resuspended and then incubated for 15 min in the dark at room temperature. After incubation, 1 μL of quencher reagent was added to every 10 μL of antibody used followed by gentle mixing and then incubation for 5 min. The subsequent mixture was stored until further use at -20°C.

### Screening of aptamers against SARS-CoV-2 S glycoprotein using direct ELONA

Direct ELONA was used to screen two aptamer sequences (CoV2-RBD-1C and CoV2-RBD-4C) against SARS-CoV-2 S protein. This technique was adopted from Sypabekova et al. (2017) with slight modifications. In brief, ninety-six well plates (M4561-40EA, Sigma-Aldrich, USA) were coated with 500 ng/mL of S protein in a 100 μL of 100 mM Na_2_CO_3_ (791768-1KG, Sigma-Aldrich, USA) buffer (pH 9.5) for 13 h at 4°C. After washing 3 times with 1×TBS (94158-10TAB, Sigma-Aldrich, USA) to remove the unbound proteins, the wells were blocked with 5% BSA (BPE1600-100, Thermo Fisher Scientific, UK) for 1 h at 4°C followed by another washing step with 1×TBS. 100 μL of biotinylated aptamers (1,500 nM) were added to each well and incubated for 2 h at 37°C followed by washing the wells 3 times with 1×TBS. A 100 μL of streptavidin-horseradish peroxidase conjugate (sHRP) (N504, Thermo Fisher, USA) was diluted in 1:10,000 in 1 × TBS and incubated for 15 min at 37°C. A 100 µL of deionized H_2_O was then added followed by the addition of a 50 µL of TMB ultrasensitive peroxidase substrate (ab142042, Abcam, UK) and incubation for 30 min at 37°C. The reaction was quenched by adding a 50 µL of 1 M H_2_SO_4_. The absorbance of wells was measured at 450 nm using an ELx800 Microplate Reader (BioTek^®^ Instruments, Inc. USA). Biotinylated SARS-CoV-2 S antibody, chimeric MAb in a concentration of 30 ng/mL served as the positive control, while in the negative control S protein was coated without aptamer, and for the background control, nuclease-free H_2_O (W4502-1L, Sigma-Aldrich, UK) was used without the S protein and an aptamer. For each sample, the absorbance (OD_450_) of background control was subtracted. All solutions were prepared using deionized water (0.055 µS/cm or 18.2 MΩxcm – 1 resistivity and 20.4°C) obtained from Barnstead™ Smart2Pure™ Water Purification System (Thermo Scientific, USA). Next, a concentration-dependent analysis for the binding affinity of CoV2-RBD-1C aptamer was performed to determine the optimal aptamer detection concentration using the following range of concentrations: 62.5, 125, 250, 500, 750, 1000, 1250, 1500, 1750, and 2000 nM using the protocol described above.

### Detection of SARS-CoV-2 S glycoprotein in buffer and human nasal fluid using direct ELONA

The detection of SARS-CoV-2 S protein in the following range of concentrations of 0, 5, 50, 500, and 5000 ng/mL diluted in a 100 mM Na_2_CO_3_ and 0.1% human nasal fluid (IRHUNFSVIAL-34884-04, Innovative Research, Netherland) using a 1,500 nM CoV2-RBD-1C aptamer was conducted. The method used for the detection of the target in buffer solution and human nasal fluid was the same as described in the screening of aptamers against SARS-CoV-2 S glycoprotein using direct ELONA section. However, for the spiked sample, human nasal fluid diluted in a 1:1000 ratio in a 100 mM Na_2_CO_3_ served as the background control, and for each sample, the absorbance (OD_450_) of the background control was subtracted.

### Specificity study

The specificity study of the ELONA technique was investigated using MERS-CoV recombinant S glycoprotein, SARS-CoV-2 N protein, influenza HA peptide, and HSA. 500 ng/mL of each non-target protein were coated overnight and further complexed with 1,500 nM of CoV2-RBD-1C aptamer. All steps used for the specificity study were the same as described in the screening of aptamers against SARS-CoV-2 S glycoprotein using direct ELONA section.

### Detection of inactivated SARS-CoV-2 variants using an aptamer-based direct ELONA

Three heat-inactivated SARS-CoV-2 variants propagated in Vero E6 cells i) hCoV-19/Kazakhstan/KazNAU-NSCEDI-Kaissar/2021 SARS-CoV-2 (Alpha variant), ii) hCoV-19/Kazakhstan/KazNAU-NSCEDI-4635/2020 SARS-CoV-2 (Wuhan variant), and iii) hCoV-19/Kazakhstan/KazNARU-NSCEDI-5526/2021 SARS-CoV-2 (Delta variant) were tested using the direct ELONA method as described in the screening of aptamers against SARS-CoV-2 S glycoprotein using direct ELONA section. The stock viral titer of 5.32 ± 0.13 Log_10_ TCID_50_/mL (Alpha), 6.20 ± 0.00 Log_10_ TCID_50_/mL (Wuhan), and 6.45 ± 0.16 Log_10_ TCID_50_/mL (Delta) were centrifuged at 5,800 rpm for 15 mins at 4°C (Chand et al., 2009) using an Eppendorf 5415 R centrifuge (Germany), and the sample was then serially diluted using a 100 mM Na_2_CO_3_ to 10^1^ to 10^5^ TCID_50_/mL and then coated onto a 96 well plate overnight at 37°C for 13 h. Subsequent steps were the same as described in the screening of aptamers against SARS-CoV-2 S glycoprotein using direct ELONA. In the negative control well, the inactivated viral samples were coated without the aptamer, while nuclease-free H_2_O served as the background control. The background absorbance (OD_450_) was also subtracted from the absorbance of all viral samples tested. The study involving the use of inactivated SARS-CoV-2 variants (BSL-1) was reviewed and approved by the biological and chemical safety committee of Nazarbayev University (42/04042022, May 25, 2022).

### Detection of inactivated SARS-CoV-2 variants in buffer and human nasal fluid using sandwich ELONA

The sandwich ELONA technique was adopted from Lee and Zeng (2017); Kim and Lee (2021); Torres-Vázquez et al. (2022), and Li et al. (2021) with modifications. In brief, a streptavidin-coated plate was made in-house by coating a 96-well plate with a 150 μL of 10 μg/mL of Pierce ™ Streptavidin (21125, Thermo Scientific, Germany) diluted in 10 mM PBS (P4417-100TAB, Sigma-Aldrich, USA) (pH 7.6) and incubating overnight at 4°C for 13 h. The wells were then washed 3 times using 1×TBS washing buffer to remove the unbound streptavidin, then, a 100 μL of 1,500 nM biotinylated CoV2-RBD-4C capture aptamer was added to each well and incubated for 2 h at 37°C. The blocking and washing steps were the same as described in the above sections. The inactivated viral samples were prepared in the same manner as described above in the detection of inactivated SARS-CoV-2 variants using an aptamer based direct ELONA section. 10^1^ – 10^5^ TCID_50_/mL concentration of the inactivated viruses were added to the 96 well plate and incubated for 1.5 h at room temperature. After incubation, subsequent steps including addition of 1,500 nM biotinylated CoV2-RBD-1C detection aptamer, incubation, washing steps and addition of s-HRP and TMB solutions were the same as described in the screening of aptamers against SARS-CoV-2 S glycoprotein using direct ELONA section. In the negative control well, the inactivated viral samples were coated without the aptamer, while nuclease free H_2_O served as the background control. The background absorbance (OD_450_) was also subtracted from the absorbance of all viral samples tested.

The detection of inactivated Wuhan and Alpha variants of SARS-CoV-2 spiked in 0.1% human nasal fluid using sandwich ELONA followed the same steps as described above, the only difference was that the 10-fold serial dilutions of the viral samples were prepared using 0.1% human nasal fluid diluted in a 100 mM Na_2_CO_3_ buffer.

### Scanning electron microscopy

Heat-inactivated Delta variant of SARS-CoV-2 was diluted in 10 mM PBS (pH 7.6) to three concentrations (6.45 × 10^3^, 6.45 × 10^4^, and 6.45 × 10^5^ TCID_50_/mL). The method for sample preparation was adopted from Haddad et al. (2020) with slight modifications. The following steps were carried out in the fume hood. All samples were fixed by adding 2.5% glutaraldehyde (G5882, Sigma-Aldrich, USA) to the sample (1:1) for 1 h. 40 µL of the fixed sample was deposited on the pure carbon films (01840-F, Ted Pella Inc, USA) for 30 min, and a filter paper was used to carefully remove the excess liquid. The grids were then immediately stained with 1% osmium tetroxide (75632, Sigma-Aldrich, USA) and allowed to dry at room temperature. The three negatively stained carbon grids were then placed on the microscope stub using a double-sided adhesive tape and sputtered with a 10 nm-thick gold layer using a turbomolecular pumped coater Q150T ES (Quorum Technologies, UK) for 10 min, to reduce charging of the non-conductive samples. The micrographs were acquired using the scanning electron microscope crossbeam 540 (Carl Zeiss, Germany). The SEM allowed to observe samples under high vacuum pressure, and the images were acquired at magnifications ranging from 5 kX to 200 kX using an electron high tension (EHT) of 5 kV and a high-resolution energy selective backscattered (ESB) electron detector.

### Data analysis

All data generated were analyzed using GraphPad Prism 9 (GraphPad Software, Inc). The one-way ANOVA followed by Bonferroni *post hoc* multiple comparison test was used to determine the statistical significance between SARS-CoV-2 RBD S glycoprotein and the non-target proteins in the specificity study, as well as the significant difference between the tested viral titers for the three heat-inactivated SARS-CoV-2 variants. The difference was considered significant when the *p*-value was less than 0.05 (*p*< 0.05). Image J software (U. S. National Institutes of Health, Bethesda, Maryland, USA) was used to process SEM micrographs and for viral particle size analysis.

## Results

### Screening of aptamers against SARS-CoV-2 S glycoprotein

**Figure 2:**
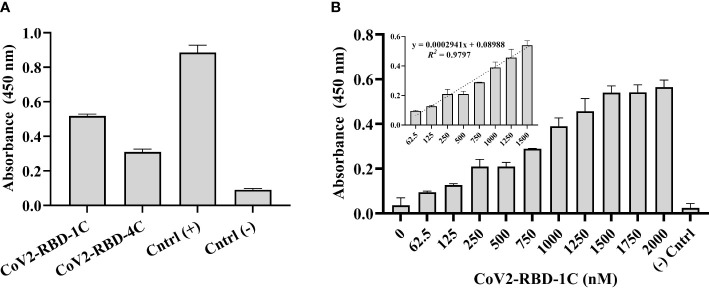
Screening of biotinylated aptamers binding affinity against SARS-CoV-2 S glycoprotein. **(A)** Direct ELONA was used to screen two aptamer sequences, CoV2-RBD-1C and CoV2-RBD-4C (1,500 nM), for the detection of S glycoprotein (N = 3). **(B)** Concentration-dependent analysis of the binding affinity of CoV2-RBD-1C aptamer sequence to SARS-CoV-2 S glycoprotein using direct ELONA (N = 3). The positive control was biotinylated SARS-CoV-2 S monoclonal antibody (30 ng/ml), RBD S glycoprotein coated with no aptamer served as the negative control, and nuclease-free water served as the background control. OD_450_ was obtained by subtracting the background control from all other wells. The *R^2^* value (**B inset**) showed that 97% of the absorbance values fitted the regression model. The regression equation for the difference in absorbance value and aptamer concentration (62.5 – 1,500 nM) was *y* = 0.0002941*x* + 0.08988, where *x* is the concentration of the aptamer in nM, and *y* is the absorbance value (OD_450_) (**B inset**). All results are represented in ± SEMs.

500 ng/mL of SARS-CoV-2 S glycoprotein was coated in a 96-well plate overnight and complexed with 1,500 nM biotinylated CoV2-RBD-1C and CoV2-RBD-4C aptamers separately to screen the sensitivity of aptamers against the target protein. Biotinylated SARS-CoV-2 spike antibody, chimeric MAb served as the positive control, while 500 ng/mL S glycoprotein coated with no aptamer served as the negative control. The absorbance at 450 nm was obtained by subtracting the optical density (OD_450_) of the background wells (nuclease-free H_2_O) from all other samples tested. Aptamer sequence CoV2-RBD-1C and CoV2-RBD-4C yielded a response signal of 0.518 ± 0.014 and 0.309 ± 0.034 at OD_450_, respectively, indicating that the aptamer sequence CoV2-RBD-1C had a better binding response (**Figure 2A**). Further, a concentration-dependent analysis of the CoV2-RBD-1C aptamer sequence was conducted in the concentration range of 62.5 - 2,000 nM using a 500 ng/mL SARS-CoV-2 S glycoprotein (**Figure 2B**). A linear correlation was found between the concentration of CoV2-RBD-1C aptamer from 62.5 to 1,500 nM and the absorbance (OD_450_) (**Figure 2B** inset). Above 1,500 nM, no further increase in OD_450_ was seen, showing that the surface was saturated above this concentration. In subsequent experiments, 1,500 nM was used as the optimal detecting concentration for CoV2-RBD-1C aptamer.

### Detection of SARS-CoV-2 S glycoprotein in buffer and human nasal fluid

**Figure 3:**
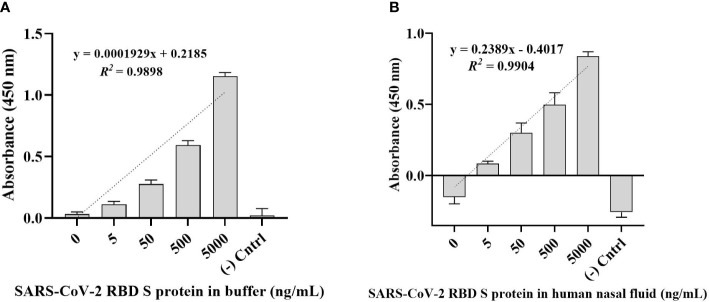
Detection of SARS-CoV-2 RBD S glycoprotein using CoV2-RBD-1C aptamer-based direct ELONA. **(A)** SARS-CoV-2 RBD S glycoprotein in a buffer solution (N = 3). Nuclease-free water was used for the background control, while S glycoprotein coated with no aptamer served as the negative control. The regression equation for difference in absorbance value and S glycoprotein concentration (5 - 5,000 ng/ml) in sodium carbonate buffer was *y* = 0.0001929*x* + 0.2185 with *R^2^* = 0.98, where *x* is the concentration of the S glycoprotein in ng/ml, and *y* is the absorbance value (OD_450_). **(B)** SARS-CoV-2 RBD S glycoprotein spiked in a 0.1% human nasal fluid (N = 6). Diluted human nasal fluid was used as the background control, while S glycoprotein in a 0.1% human nasal fluid coated with no aptamer served as the negative control. Absorbance was obtained by subtracting the background control from all other wells. The regression equation for OD_450_ difference versus S glycoprotein concentration (0 - 5,000 ng/ml) in 0.1% human nasal fluid was *y* = 0.2389*x* – 0.4017 with *R^2^* = 0.99. All results are presented in ± SEMs.

The direct ELONA method was used for the detection of SARS-CoV-2 S glycoprotein in a Na_2_CO_3_ buffer solution in the concentration range from 5 - 5,000 ng/mL (**Figure 3A**). RBD S protein was also spiked in a 0.1% human nasal fluid in the same concentration range (**Figure 3B**) using 1,500 nM optimal detection concentration of CoV2-RBD-1C aptamer. Specifically, we obtained an OD_450_ for SARS-CoV-2 RBD S protein in buffer solution at 500 ng/mL to be 0.592 ± 0.051 (N = 3) while for 500 ng/mL RBD S protein spiked in 0.1% human nasal fluid yielded an OD_450_ of 0.533 ± 0.144 (N = 6). A linear correlation was found between OD_450_ value and S glycoprotein in a range of concentrations from 5 to 5,000 ng/ml (**Figures 3A, B** **)**.

The limit of detection (LOD) was calculated using $LOD=\frac{3.3\;(Sy.x)}{Slope}$ where *Sy.x* is the standard deviation of the linear regression (Shrivastava and Gupta, 2011). As a result, the calculated LOD for SARS-CoV-2 RBD S glycoprotein in a buffer solution was 2.16 ng/mL, while the LOD for SARS-CoV-2 RBD S protein in a 0.1% human nasal fluid was 1.02 ng/mL in a 100 µL volume of the sample.

### Specificity study

**Figure 4:**
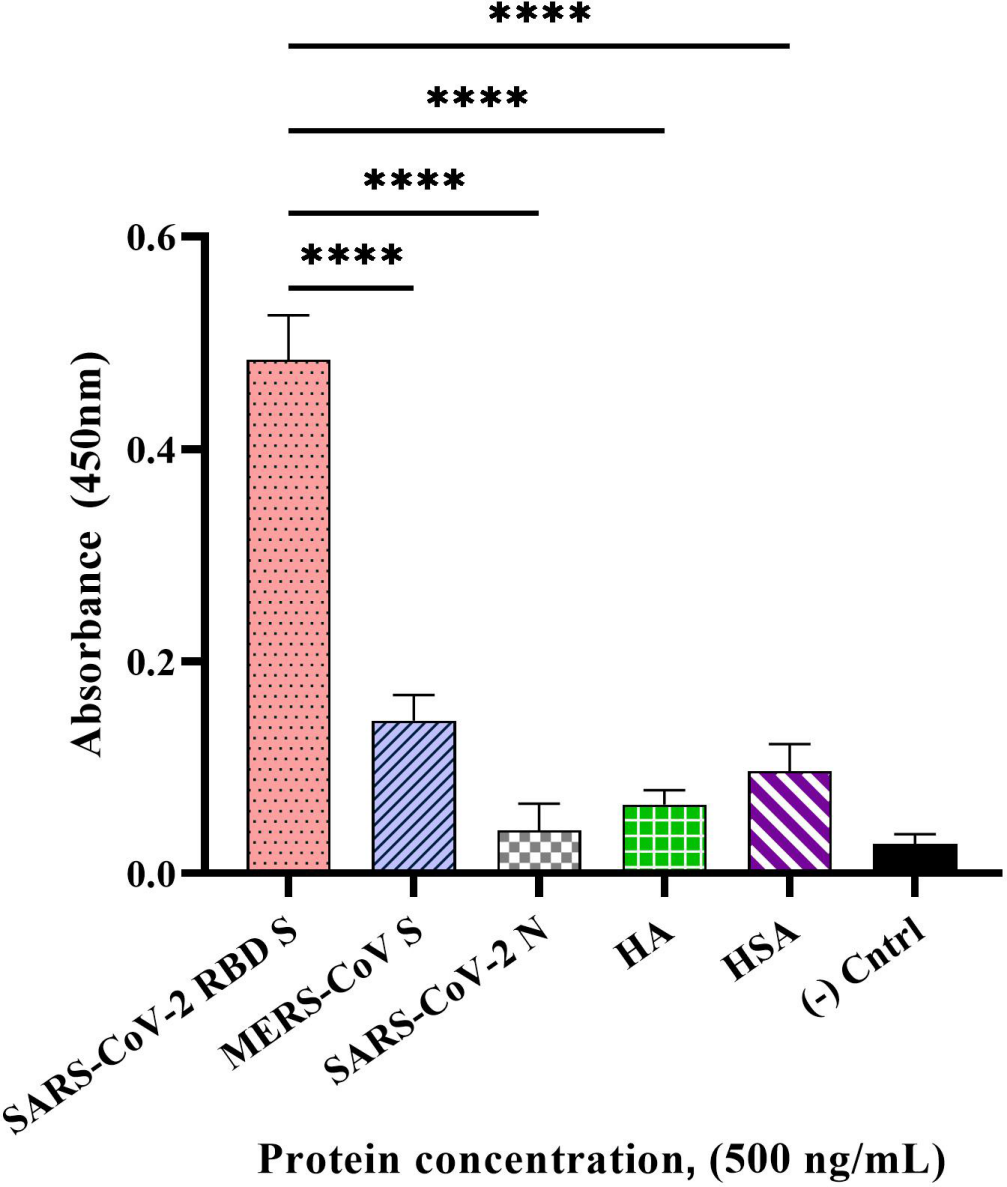
Specificity of the direct ELONA to SARS-CoV-2 S glycoprotein. Non-target proteins, MERS-CoV S glycoprotein, SARS-CoV-2 N protein, HA peptide, and HSA at a concentration of 500 ng/mL coupled with the CoV2-RBD-1C aptamer (1,500 nM) were employed in this study (N = 3). Nuclease-free water was used for the background control, while SARS-CoV-2 S glycoprotein coated with no aptamer served as the negative control. Absorbance was obtained by subtracting the background control from all other wells. All results are presented in ± SEMs. ****p< 0.0001.

The specificity of our developed direct ELONA using CoV2-RBD-1C aptamer sequence at an optimal concentration of 1,500 nM was tested with the following non-target proteins at 500 ng/mL concentration: MERS-CoV S, SARS-CoV-2 N, influenza HA peptide, and HSA. The OD_450_ was obtained by subtracting the background from sample wells. We obtained the following OD_450_ for the samples tested, 0.484 ± 0.082 for SARS-CoV-2 S glycoprotein, 0.144 ± 0.026 for MERS-CoV S glycoprotein, 0.040 ± 0.011 for SARS-CoV-2 N protein, 0.065 ± 0.053 for HA and 0.096 ± 0.046 for HSA (**Figure 4**). We found a statistically significant difference between the target S glycoprotein and the four non-target proteins tested with *p*< 0.0001 for each of the comparisons.

### Detection of inactivated SARS-CoV-2 variants in buffer using direct ELONA

**Figure 5:**
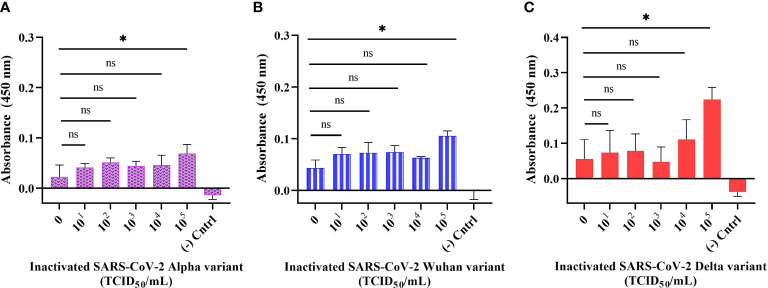
Detection of inactivated SARS-CoV-2 variants in buffer using direct ELONA. Inactivated SARS-CoV-2 **(A)** Alpha, **(B)** Wuhan, and **(C)** Delta variants. Nuclease-free water was used for the background control, while the corresponding inactivated variants without aptamer served as the negative control. Absorbance was obtained by subtracting the background control from all other wells where the viral samples were tested. All results are presented in ± SEMs (N = 3), and the statistically significant differences between each viral titer were determined: ns, not significant, *p< 0.05.

The sensitivity of the developed direct ELONA was validated using inactivated SARS-CoV-2 samples. We tested Alpha, Wuhan, and Delta variants of SARS-CoV-2 in a viral titer range in TCID_50_/mL (Median Tissue Culture Infectious Dose) as follows: for Alpha variant 5.32 × 10^1^ to 5.32 × 10^5^ TCID_50_/mL, for Wuhan variant 6.20 × 10^1^ to 6.20 × 10^5^ TCID_50_/mL, and for Delta variant the viral titer range was from 6.45 × 10^1^ to 6.45 × 10^5^ TCID_50_/mL. The data obtained for the direct ELONA (**Figure 5**) indicates that the direct ELONA using CoV2-RBD-1C aptamer was able to detect Alpha, Wuhan as well as Delta variants of SARS-CoV-2 from 10^5^ TCID_50_/mL, which was statistically significant from 0 TCID_50_/mL (buffer) with *p*< 0.05 (**Figures 5A-C**). From the results, our developed method was able to detect the Alpha variant at a specific viral titer from 5.32 × 10^5^ TCID50/mL with an OD_450_ of 0.069 ± 0.025, Wuhan variant from 6.20 × 10^5^ TCID_50_/mL with an OD_450_ of 0.105 ± 0.021, and 6.45× 10^5^ TCID_50_/mL for the Delta variant with an OD_450_ of 0.224 ± 0.038 (**Figure 5**). 0 TCID_50_/mL represents the dilution buffer (Na_2_CO_3_) without the viral samples. In the negative control well, the inactivated viral samples were coated without the aptamer, while nuclease-free H_2_O served as the background control. The absorbance (OD_450_) was obtained by subtracting the background from the absorbance of all viral samples tested. The specific viral titer detected for the three variants was converted to PFU/mL using the standard conversion (Mena et al., 2003), $\frac{PFU}{mL}=\frac{TCID50}{mL}\times \;0.69.$ We converted TCID_50_/mL to PFU/mL and as a result, the detected viral titer in PFU/mL for the three variants as suggested from **Figure 5** were 3.67 × 10^5^ PFU/mL for Alpha variant, 4.28 × 10^5^ PFU/mL for Wuhan variant, and 4.45 × 10^5^ PFU/mL for Delta variant. The limit of detection obtained for our developed direct ELONA were 3.73, 5.72, and 6.02 TCID_50_/mL in a 100 µL volume of inactivated Alpha, Wuhan, and Delta variants of SARS-CoV-2, respectively. These findings agrees with Lescure et al. (2020), where they measured the viral titer in the nasopharyngeal swab of two SARS-CoV-2 positive patients, and reported the viral isolate titer to be 6.25 × 10^5^ and 3.0 × 10^7^ PFU/mL, in addition, an electrochemical immunosensor also detected SARS-CoV-2 in a detection limit from 13.75 × 10^5^ and 5.5 × 10^5^ PFU/mL (Mojsoska et al., 2021).

### Detection of inactivated SARS-CoV-2 variants in buffer using sandwich ELONA

**Figure 6:**
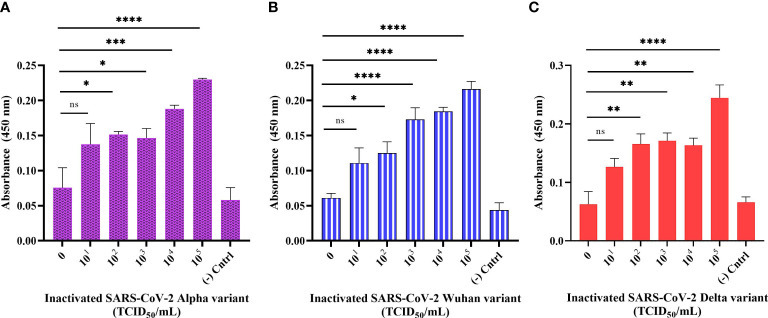
Detection of inactivated SARS-CoV-2 variants in buffer using sandwich ELONA. Inactivated SARS-CoV-2 **(A)** Alpha, **(B)** Wuhan, and **(C)** Delta variants. Nuclease-free water was used for the background control, while the corresponding inactivated variants without aptamer served as the negative control. Absorbance was obtained by subtracting the background control from all other wells where the viral samples were tested. All results are presented in ± SEMs (N = 3), and the statistically significant differences between each viral titer was determined: ns - not significant, *p< 0.05, **p< 0.01, ***p< 0.001, ****p< 0.0001. The regression equation for difference in absorbance value versus Alpha, Wuhan, and Delta variants (0 to 10^5^ TCID_50_/mL) was *y* = 0.02625*x* + 0.08916, *y* = 0.02988*x* + 0.07037, and *y* = 0.02928*x* + 0.08237 with *R^2^* = 0.89, 0.97, and 0.84 for the three variants, respectively.

We designed a sandwich ELONA to further enhance the sensitivity signal for the detection of SARS-CoV-2 by employing two aptamer sequences, CoV2-RBD-4C and CoV2-RBD-1C (**Figure 1**). The wells were first pre-coated with streptavidin because the interaction between biotin and streptavidin is the strongest non-covalent binding (Chivers et al., 2011), and further inactivated Alpha, Wuhan, and Delta variants were captured using a biotin-tagged CoV2-RBD-4C aptamer and detected using biotin tagged COV2-RBD-1C aptamer followed by an enzyme-labeled substrate (s-HRP) and addition of TMB substrate (Platella et al., 2017). This sandwich ELONA system detected the three inactivated variants of SARS-CoV-2 in a concentration-dependent manner. Our developed method was able to detect the Alpha variant in a viral titer as low as 5.32 × 10^2^ TCID_50_/mL with an OD_450_ of 0.151 ± 0.021, Wuhan variant from 6.20 × 10^2^ TCID_50_/mL with an OD_450_ of 0.125 ± 0.027, and 6.45× 10^2^ TCID_50_/mL for the Delta variant with an OD_450_ of 0.165 ± 0.022 in a buffer (**Figures 6A–C**). LODs were calculated using an equation as specified above, where we obtained 2.31, 1.15, and 2.96 TCID_50_/mL in a 100 µL volume of inactivated Alpha, Wuhan, and Delta variants, respectively, which were significantly lower than the LOD obtained in the direct ELONA. 0 TCID_50_/mL represents the dilution buffer (Na_2_CO_3_) without the viral samples. In the negative control well, the inactivated viral samples were coated without the aptamer, while nuclease free H_2_O served as the background control. The absorbance (OD_450_) was obtained by subtracting the background from the absorbance of all viral samples tested. Further analysis shows that a linear correlation was found between OD_450_ and the three variants of SARS-CoV-2 in a range of concentration from 0 to 10^5^ TCID_50_/mL.

### Detection of inactivated SARS-CoV-2 variants spiked in a human nasal fluid using sandwich ELONA

**Figure 7:**
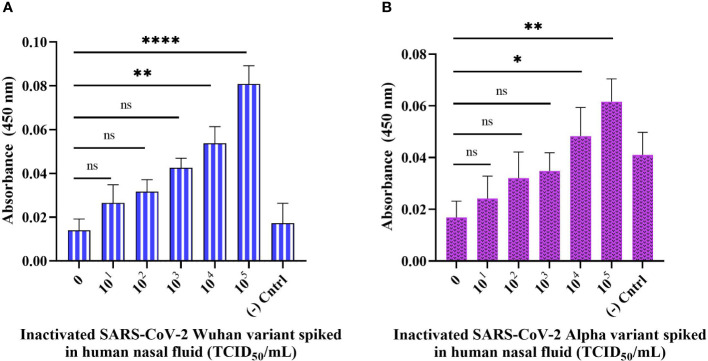
Detection of inactivated SARS-CoV-2 variants spiked in a 0.1% human nasal fluid using sandwich ELONA. Inactivated SARS-CoV-2 **(A)** Wuhan variant, and **(B)** Alpha variant. 0.1% nasal fluid without inactivated viral samples was used as the background control, while each inactivated variant without the aptamers served as the negative control. Absorbance was obtained by subtracting the background control from all other wells where the viral samples were coated. All data are presented in ± SEMs (N = 6), and the statistically significant differences between the viral titer tested was determined: ns – not significant, *p< 0.05, **p< 0.01, ****p< 0.0001. The regression equation for difference in absorbance value and Wuhan variant viral titer (0 to 10^5^ TCID_50_/mL) in 0.1% human nasal fluid was *y* = 0.01219*x* + 0.01106 with *R^2^* = 0.93, while for Alpha variant the regression equation was *y* = 0.008557*x* + 0.01491 with *R^2^* = 0.95, where *x* is the concentration of the inactivated variants in TCID_50_/mL, and *y* is the absorbance value (OD_450_).

To test the practicality of the developed sandwich assay in a clinical setting, we validated the sandwich ELONA system with spiked samples i.e., inactivated viral samples diluted in a 0.1% human nasal fluid and tested in a concentration-dependent manner. In this experiment, Wuhan and Alpha variants of SARS-CoV-2 were spiked into a 0.1% human nasal fluid and the viral titer tested in a concentration-dependent manner for the Wuhan variant was from 6.20 × 10^1^ to 6.20 × 10^5^ TCID_50_/mL, while for Alpha variant it was from 5.32 × 10^1^ to 5.32 × 10^5^ TCID_50_/mL (**Figure 7A, B** **)**. Diluted human nasal fluid without the viral samples and aptamers served as the background control, while the viral samples were immobilized in the negative control well without the aptamers. OD_450_ was obtained by subtracting the absorbance of the background control from the OD_450_ obtained for all viral samples tested. From **Figure 7A**, the sandwich ELONA detected the spiked Wuhan variants sample in a viral titer from 6.20 × 10^4^ TCID_50_/mL having an OD_450_ of 0.053 ± 0.02 with a LOD of 1.79 TCID_50_/mL. We also observed that the Alpha variant was detected from 5.32 × 10^4^ TCID_50_/mL viral titer having an OD_450_ of 0.0483 ± 0.00168 with a LOD of 1.41 TCID_50_/mL (**Figure 7B**). The result shows that the interaction between inactivated variants of SARS-CoV-2 with both capture and reporter aptamers (**Figures 7A, B** **)** can be applied in this novel SARS-CoV-2 sandwich ELONA detection system. A linear correlation was found between OD_450_ and inactivated variants of SARS-CoV-2 in a range of concentrations from 0 to 10^5^ TCID_50_/mL.

### SEM study

**Figure 8:**
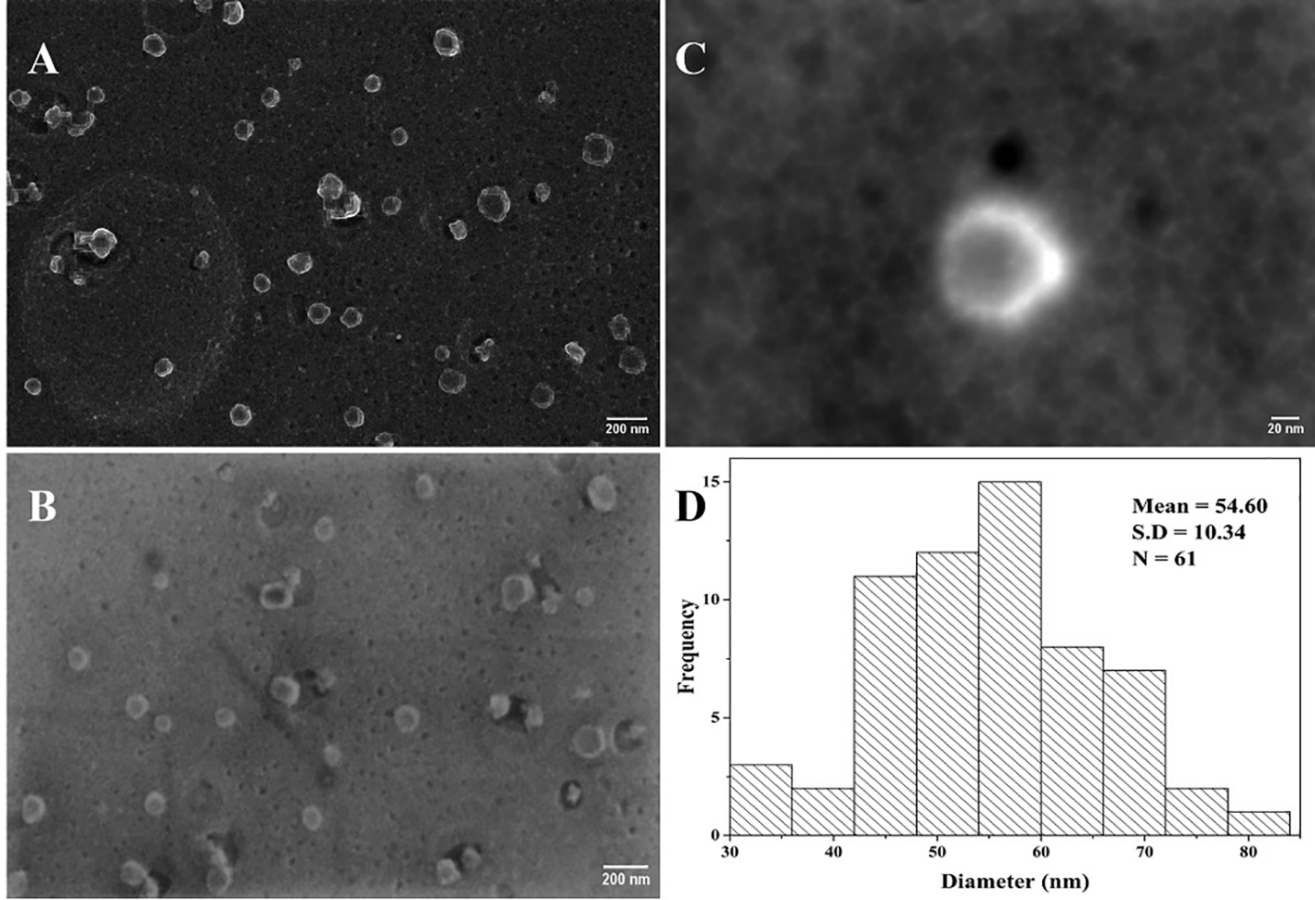
Heat-inactivated Delta variant SEM micrographs of SARS-CoV-2. **(A, B)** Viral particle distribution on a carbon grid (scale bar = 200 nm); **(C)** inactivated viral particle with round and shiny crownlike edges of SARS-CoV-2 (scale bar = 20 nm); **(D)** histogram showing the corresponding size distribution of **(A)** and **(B)** micrographs (N = 61).

The objective of the SEM study was to visualize the presence of viral particles in the heat-inactivated SARS-CoV-2 sample. This proof-of-concept SEM study was carried out using the Delta variant of the virus. High-resolution micrographs (**Figures 8A–C**) were acquired from Carl Zeiss Crossbeam 540 SEM (Germany) having a clear distribution of viral particles at a viral titer level of 10^4^ TCID_50_/mL. The viral particle as seen on the micrograph (**Figure 8C**) shows an electron-dense spherical particle surrounded by a shiny crown-like shape projection known as the spike. Each SARS-CoV-2 viral particle were selected manually from **Figures 8A, B**, and the diameter was measured after converting the threshold images into binary (Barreto-Vieira et al., 2022). After measurement, the size distribution data of the viral particles were plotted on a histogram (**Figure 8D**) using the OriginLab analysis software (OriginLab Corporation, Northampton, MA, USA). The viral particle diameter was measured using the ImageJ software and we selected manually 61 viral particles (**Figures 8A, B** **)**. The sizes ranged from 33.5 to 81 nm with an average diameter of 54.60 nm.

## Discussion and conclusion

ELISA has been routinely used for the diagnosis of SARS-CoV-2 infection frequently involving the use of antibodies (Lee and Zeng, 2017). Aptamers, however, can succeed in replacing antibodies especially when specific and effective binding to a target is required. This study demonstrates that an aptamer-based ELONA can successfully detect SARS-CoV-2, which is not only simple to run but also sensitive. Here, we screened the efficiency of the two aptamer sequences against SARS-CoV-2 RBD S glycoprotein using ELONA for the sole purpose of developing a simple and sensitive detection system. We found that the aptamer sequence CoV2-RBD-1C had a better binding response to the S glycoprotein (**Figure 2A**), which agrees with the findings of Song and his group (2020), where the *K_D_* values of CoV2-RBD-1C and CoV2-RBD-4C aptamers against S glycoprotein was 5.8 nM and 19.9 nM, respectively, suggesting better binding kinetics for CoV2-RBD-1C aptamer. In addition, a high OD_450_ was obtained in the positive control (**Figure 2A**), which may be attributed to steric hindrance and size differences between aptamer and antibody. The small size of an aptamer is favorable for immobilization, stability at various temperature fluctuations, tolerance to stringent chemical and biological conditions, and penetration/binding with complex proteins (Bauer et al., 2019). Despite the absorbance of antibodies obtained from our ELONA, the use of antibodies might be detrimental for long-term storage, chemical alterations, and batch-to-batch differences (Lakhin et al., 2013). CoV2-RBD-1C aptamer detection concentration was optimized, and the result suggested an optimal detection concentration at 1,500 nM (**Figure 2B**). The absorbance was measured at 450 nm wavelength where TMB has the highest absorbance peak.

To test the sample matrix influence on the sensitivity of the direct ELONA, SARS-CoV-2 RBD S glycoprotein was spiked into a 0.1% of commercially available human nasal fluid (**Figure 3**). Clinical human nasal fluid influenced S glycoprotein detection compared to a buffer-based S glycoprotein. This may be attributed to the presence of glycoproteins such as mucins and other proteins in human nasal fluid, which are also rich in ions (Na^+^, K^+^, Cl^-^, and Ca^2+^) that are generated by mucous gland cells (Vanthanouvong and Roomans, 2004).

The specificity of our detection system was further validated using four non-target proteins: MERS-CoV S glycoprotein, SARS-CoV-2 N protein, influenza HA peptide, and HSA (**Figure 4**). MERS-CoV belongs to the same genera of β-CoV and has a similar S glycoprotein to SARS-CoV-2 that mediates viral attachment and infection in the host cell and a genome size of about 30,000 nucleotides (Chong et al., 2021). SARS-CoV-2 encodes the N structural protein, and SARS-CoV-2 infection causes viral genomic RNA to bind to the N protein, generating a ribonucleoprotein complex, which folds into a helical shape and attaches to the viral N protein. Infected patients demonstrated a greater and earlier humoral response to the N protein than to the S glycoprotein, despite the N protein being enclosed within the viral particle (Khan et al., 2022). Influenza HA peptide was also employed since it infects respiratory tract cells and uses surface proteins to infect host cells, in the instance of SARS-CoV-2, the S glycoprotein aids viral entrance and infection in the host cell (Manzanares-Meza and Medina-Contreras, 2020). HSA is the most abundant circulating plasma protein in humans and is sensitive to non-enzymatic glycation (Rabbani and Ahn, 2021). Albumin inhibits SARS-CoV-2 receptor ACE2 and hypoalbuminemia, coagulopathy, and vascular disease appear to predict coronavirus outcomes independent of age or morbidity (Rabbani and Ahn, 2021). HSA is a valuable biomarker of numerous diseases, including SARS-CoV-2 (Turcato et al., 2022). Planned comparisons between the OD_450_ obtained by ELONA for SARS-CoV-2 S glycoprotein, SARS-CoV-2 N protein, MERS-CoV S glycoprotein, influenza HA, and HSA suggest a high specificity to SARS-CoV-2 S glycoprotein.

Furthermore, we proposed that since Song and his group have shown the binding interface of CoV2-RBD-1C and CoV2-RBD-4C creates a strong hydrogen bond with RBD’s amino acids: Thr500, Gln506, Asn437, Gln409, Lys417, and Tyr421 through molecular docking and molecular dynamics (Song et al., 2020), then our developed aptamer-based ELONA technique could detect Alpha, Wuhan, and Delta variants of SARS-CoV-2 since the variants still maintained some of the amino acids that are involved in the binding between CoV2-RBD-1C, CoV2-RBD-4C, and the RBD S glycoprotein of SARS-CoV-2. To validate the practical application of the developed assay, we employed three heat-inactivated variants, i) the Alpha variant, which was obtained from the nasopharyngeal swab of a 39-year-old male from Almaty, Kazakhstan (GISAID accession no. EPI_ISL_2349552) which has about 9 mutations in the S glycoprotein, ii) the Delta variant that was obtained from the nasopharyngeal swab of a 31-year-old female Zhanaozen, Kazakhstan having about 12 mutations found in the S glycoprotein (GISAID accession no. EPI_ISL_4198501), iii) while the Wuhan variant was also obtained from the nasopharyngeal swab of a 31-year-old male from Almaty, Kazakhstan having only D614G and M153T mutations in the S glycoprotein (GISAID accession no. EPI_ISL_1208952) (Tabynov et al., 2022). Our aptamer based ELONA technique presented a good detection absorbance (**Figures 5**, **6**, **7**) due to the retention of the amino acids in the three variants that creates a strong hydrogen bond with the aptamers employed in this study, for example, N501Y mutation of Alpha expressed an asparagine (Asn) which was replaced with tyrosine (Tyr) at position 501 (Socher et al., 2022), while in the Wuhan variant, M153T mutation expressed methionine (Met) which was replaced with threonine (Thr) at position 153, and in the Delta variant, D950N mutation expressed aspartate (Asp) that was replaced with asparagine (Asn) at position 950.

According to a study conducted by Zou et al. (2020), heat inactivation of samples decreases the RT-PCR detection rates of SARS-CoV-2 thereby leading to a large number of false negative results, which could be as a result of the loss or displacement of the S glycoprotein projections (Prasad et al., 2021). This explains the low absorbance signal (OD_450_) for the viral titer tested from 10^1^ to 10^4^ TCID_50_/mL in the direct ELONA and also could be due to the presence of fetal bovine serum and other substances like antibiotics present in the DMEM culture medium (Choi et al., 2014), where the variants of SARS-CoV-2 were propagated, and can potentially affect the detection signal. A study also reported that high centrifugal speeds generated by fixed angle centrifuge rotors allow the precipitate to concentrate along the vertical axis of the sample tube, making it less visible and difficult to reconstitute, potentially resulting in viral precipitate loss and reduced yields (Engler and Selepak, 1994; Mailepessov et al., 2022). As a result of that, centrifugation of the samples could also be a reason for low absorbance signal due to loss of viral precipitate or reduced viral loads. Furthermore, we observed that it is important to subject the samples to preconcentration based on centrifugation because in our experiment without the centrifugation step, the absorbance was lower than when the samples were centrifuged (data not shown).

The sensitivity of the developed assay does well in comparison with other studies described in the literature, where they exploited different aptamer sequences for the detection of SARS-CoV-2 as summarized in **Table 2**. Both direct and sandwich ELONA used in this study performed excellently, with similar or even improved performance compared to other SARS-CoV-2 detection methods. For example, a study reports the measurement of the viral titer in the nasopharyngeal swab of two SARS-CoV-2 positive patients to be 6.25 × 10^5^ and 3.0 × 10^7^ PFU/mL (Lescure et al., 2020), in addition, an electrochemical immunosensor also detected SARS-CoV-2 with a detection limit between 13.75 × 10^5^ and 5.5 × 10^5^ PFU/mL (Mojsoska et al., 2021), these findings are in agreement with data obtained for the direct ELONA in which the following viral titer were detected: 3.67 × 10^5^ PFU/mL for Alpha variant, 4.28 × 10^5^ PFU/mL for Wuhan variant, and 4.45 × 10^5^ PFU/mL for Delta variant, however, for the sandwich ELONA the viral titer detected for Alpha, Wuhan and Delta variants were as low as 3.67 × 10^2^ PFU/mL, 4.28 × 10^2^ PFU/mL and 4.45 × 10^2^ PFU/mL, respectively. Similarly, another study reported a sandwich ELONA for the detection of SARS-CoV-2 S1 protein with an LOD of 21 ng/mL (Svobodova et al., 2021) in buffer, however, our direct ELONA for detection SARS-CoV-2 S glycoprotein shows a better performance with an LOD of 2.16 ng/mL in buffer. As a matter of fact, the detection limit of our sandwich ELONA for the detection of heat-inactivated SARS-CoV-2 variants is also in agreement with Kacherovsky et al. (2021) where they detected UV-inactivated SARS-CoV-2 using sandwich ELISA.

**Table 2:** Colorimetric assays developed for the detection of SARS-CoV-2.

Type of detection	Target	Recognition element	Dynamic range	Limit of detection	References
Sandwich ELISA	UV-inactivated SARS-CoV-2	Aptamer (SNAP1) and HRP anti-fluorescein antibody	1 × 10^5^ – 1 × 10^7^ copies/mL	5 × 10^5^ copies/mL	(Kacherovsky et al., 2021)
ELONA	2019-nCov-NP	Aptamer (N48 and N61)	0 – 6 ng/mL	93.75 pg/mL	(Tian et al., 2021)
Sandwich assay	Pseudotyped SARS-CoV-2 spiked in 50% saliva	MSA1 aptamer	0 – 200 pM	400 fM	(Li et al., 2021)
ELISA	Pseudotyped SARS-CoV-2	C5-Fc-SS-biotin	10^1^ – 10^4^ TCID_50_/mL	16 TCID_50_/mL	(Girt et al., 2021)
	Inactivated SARS-CoV-2	C5-Fc-SS-biotin	10^1^ – 10^4^ ffu/mL	16 ffu/mL	
Direct ELONA	SARS-COV-2 spike antigen	S14 aptamer	0.5 – 125 nM	2 nM	(Gupta et al., 2021)
Sandwich ELONA	SARS-CoV-2 S1	Apt1T/Apt5B	0.001 – 100 µg/mL	21 ng/mL in buffer 15 ng/mL in VTM	(Svobodova et al., 2021)
ELISA	SARS−CoV−2 S1 antigen	Monoclonal IgG antibody	0 – 20 ng/mL	1 ng/mL	(Mades et al., 2021)
Direct ELONA	SARS-CoV-2 S glycoprotein in buffer	CoV-RBD-1C	0 – 5 μg/mL	2.16 ng/mL	This study
	SARS-CoV-2 S glycoprotein spiked in human nasal fluid	CoV-RBD-1C	0 – 5 μg/mL	1.02 ng/mL	This study
	Inactivated Alpha variant in buffer	CoV-RBD-1C	5.32 × 10^1^ – 5.32 × 10^5^ TCID_50_/mL	3.73 TCID_50_/mL	This study
	Inactivated Wuhan variant in buffer	CoV-RBD-1C	6.20 × 10^1^ – 6.20 × 10^5^ TCID_50_/mL	5.72 TCID_50_/mL	This study
	Inactivated Delta variant in buffer	CoV-RBD-1C	6.45 × 10^1^ – 6.45 × 10^5^ TCID_50_/mL	6.02 TCID_50_/mL	This study
Sandwich ELONA	Inactivated Alpha variant in buffer	CoV-RBD-4C/CoV-RBD-1C	5.32 × 10^1^ – 5.32 × 10^5^ TCID_50_/mL	2.31 TCID_50_/mL	This study
	Inactivated Wuhan variant in buffer	CoV-RBD-4C/CoV-RBD-1C	6.20 × 10^1^ – 6.20 × 10^5^ TCID_50_/mL	1.15 TCID_50_/mL	This study
	Inactivated Delta variant in buffer	CoV-RBD-4C/CoV-RBD-1C	6.45 × 10^1^ – 6.45 × 10^5^ TCID_50_/mL	2.96 TCID_50_/mL	This study
	Inactivated Alpha variant spiked in human nasal fluid	CoV-RBD-4C/CoV-RBD-1C	5.32 × 10^1^ – 5.32 × 10^5^ TCID_50_/mL	1.41 TCID_50_/mL	This study
	Inactivated Wuhan variant in human nasal fluid	CoV-RBD-4C/CoV-RBD-1C	6.20 × 10^1^ – 6.20 × 10^5^ TCID_50_/mL	1.79 TCID_50_/mL	This study

UV, ultraviolet radiation; VTM, viral transport medium; NP, nucleocapsid protein; HRP, horse radish peroxidase; dynamic range, the range of analyte concentration tested.

Furthermore, the SEM was employed to confirm the presence of the viral particles in the samples to be tested, to observe the size, and to determine whether heat inactivation contributed to the size deformation in the Delta variant of SARS-CoV-2. Despite heat-inactivation at 65°C for 30 min, we were able to visualize viral particles having a spherical shape with shining spikes on the envelopes (**Figure 8**). According to the findings of the SEM study, the average diameter of the Delta variant was 54.48 nm, this is in contrast to the 125 nm diameter reported (Neuman et al., 2006), confirming that the heat inactivation of SARS-CoV-2 viral particles leads to a size alteration (Lyonnais et al., 2021).

In conclusion, we developed a simple and sensitive ELONA that can detect three heat-inactivated variants of SARS-CoV-2 by utilizing commercially available and low-cost reagents. The direct ELONA detected SARS-CoV-2 S glycoprotein in buffer solution at 2.16 ng/mL and in 0.1% human nasal fluid at 1.02 ng/mL LOD. While the absorbance signal suggests a highly specific reaction for SARS-CoV-2 S glycoprotein, there was limited cross-reactivity with MERS-CoV S glycoprotein and low cross reaction with non-target proteins such as SARS-CoV-2 N protein, influenza HA peptide, and HSA. Heat-inactivated Alpha, Wuhan, and Delta variants of SARS-CoV-2 were detected with a LOD that is within the acceptable viral titer detection range. By adopting the sandwich configuration, we amplified the ELONA response signal and increased the sensitivity level for all three variants in 5 h sample-result time. Spiking the Alpha and Wuhan variants of SARS-CoV-2 into 0.1% human nasal fluid also produced a good detection absorbance in a viral titer similar to prior studies using our developed technique, which could potentially be adopted for detection of SARS-CoV-2 in clinical settings. Future research will focus on implementation of our developed ELONA technique with clinical samples from infected patients.

## Data availability statement

The original contributions presented in the study are included in the article/supplementary material. Further inquiries can be directed to the corresponding author.

## Author contributions

MSD: investigation, methodology, formal analysis, conceptualization, validation, writing – original draft. DK: conceptualization, methodology, visualization, supervision, project administration, funding acquisition, writing - review & editing. All authors contributed to the article and approved the submitted version.

## Funding

This research was funded by the Science Committee of the Ministry of Education and Science (MES) of the Republic of Kazakhstan (grant no. АР08053347) and Nazarbayev University (NU) (Nur-Sultan, Kazakhstan) (grant no. 280720FD1911). MSD received the Abay Kunanbayev scholarship for the MSc studies awarded by NU.

## Acknowledgments

We thank the Masgut Aikimbayev National Scientific Center for Dangerous Infections (Almaty, Kazakhstan) for providing us with the heat-inactivated variants of SARS-CoV-2. We also thank the Core Facility at NU for providing the access to the SEM and especially Nurgul Daniyeva for providing technical assistance in the SEM study.

## Conflict of interest

The authors declare that the research was conducted in the absence of any commercial or financial relationships that could be construed as a potential conflict of interest.

## Publisher’s note

All claims expressed in this article are solely those of the authors and do not necessarily represent those of their affiliated organizations, or those of the publisher, the editors and the reviewers. Any product that may be evaluated in this article, or claim that may be made by its manufacturer, is not guaranteed or endorsed by the publisher.
